# Prevalence and factors associated with high-risk human papillomavirus infection and cervical lesions among women with HIV in Addis Ababa, Ethiopia

**DOI:** 10.1186/s12885-025-14832-3

**Published:** 2025-10-13

**Authors:** Helen Negassi Tesfom, Muluken Gizaw, Mengistu Yilam, Johanna M. A. Klein, Eva J. Kantelhardt, Sefonias Getachew

**Affiliations:** 1 ICAP-Ethiopia , Addis Ababa, Ethiopia; 2 Department of Preventive Medicine, School of Public Health, Addis Ababa University , Addis Ababa, Ethiopia; 3 Global Health Working Group, Institute for Medical Epidemiology, Biometrics and Informatics (IMEBI), Martin-Luther-University Halle, Halle, 06120 Germany; 4 Department of Gynecology, Martin Luther University, Halle, Germany

**Keywords:** HPV, Genotype distribution, Cervical lesion, VIA, HIV, Ethiopia

## Abstract

**Background:**

In Ethiopia, the Human Papillomavirus (HPV) type distribution is still not well characterized among women with Human Immunodeficiency Virus (HIV). This study aimed to assess the prevalence of HPV infection, determine the distribution of HPV genotypes, and examine the rate of abnormal findings from visual inspection with acetic acid (VIA) among HPV-positive women living with HIV (WWH) in Addis Ababa, Ethiopia. It also analyzed the factors associated with these findings.

**Methodology:**

A retrospective record review was conducted at 16 HPV-DNA testing health facilities in Addis Ababa, Ethiopia. A total of 3303 WWH were screened for HPV infection through self- and clinician-based cervical swab collection from April 2021 to January 2022, all were included in the study. WWH were offered HPV testing at antiretroviral therapy (ART) clinics. Trained nurses invited women positive for high-risk HPV (hrHPV) types for VIA screening. Women with suspicious lesions were referred to gynaecologic centres, and others were appointed for a follow-up visit after one year. Associations between independent variables and HPV-DNA as well as VIA positivity were assessed using Binary logistic regression.

**Result:**

The prevalence of hrHPV infection was 28.7% (95% CI: 27.2, 30.3%). Of those, 145 (15.3%) had HPV 16, and 38 (4%) had HPV 18 and the rest larger proportion was “other high-risk HPV” accounted for 695 (73.3%). The data showed that 70 (7.4%) women had multiple hrHPV infection, meaning ≥ 2 HPV DNA types were found in a single sample. This study found that the odds of women aged 25–29 being infected with hrHPV was 68% higher than those of women aged 40–49 (Adjusted Odds Ratio (AOR) = 1.68, 95% CI 1.26, 2.22). Women who were married and widowed had lower odds of hrHPV infection as compared to divorced women. Out of 948 hrHPV-positive clients, 786 (82.9%) had a follow up VIA screening, of which 15.5% (122) were found to be VIA positive. A history of STI was significantly associated with VIA positivity (AOR = 1.69, 95% CI 1.12, 2.54).

**Conclusion:**

The prevalence of hrHPV in WWH was high as compared to other studies done in Ethiopia, particularly that of HPV 16 and “other hrHPV”. Prevalence of cervical lesion based on VIA was higher as compared to other studies that did not initially screen for hrHPV infection. This underlines the need to offer screening services to WWH and further characterize the other hrHPV. Screening for cervical lesions is of particular importance in women who report a history of STIs.

## Introduction

Globally, cervical cancer is the fourth most common cancer and the fourth leading cause of cancer death in women [1]. An estimated 662,301 new cases and 348,874 deaths occurred worldwide in 2022 [1]. The highest regional incidence and mortality is in sub-Saharan Africa, and the highest incidence of human papillomavirus (HPV) infection and cervical cancer was found to be in Eastern and Southern Africa [1, 2]. In Ethiopia, cervical cancer is the second most common cancer next to breast cancer, and the third leading cause of cancer mortality among women aged 15–44 [3]. An estimated 8168 new cases and 5975 deaths from cervical cancer occur annually in Ethiopia (estimates for 2022) [3].

Women living with human immunodeficiency virus (WWH) have a high prevalence of HPV infection resulting from inability of the host to clear the infection and reactivation of latent HPV infections due to their immune suppression [4, 5]. Evidence also suggested that WWH are more likely to have persistent HPV infections, which will result in a more rapid progression to pre-cancer and cancer [6–8].

Studies conducted in different regions of Ethiopia showed a prevalence of hrHPV in the general population ranging from 7 to 23% [9, 10]. A systematic review of the literature which included ten studies from 2005 to 2019 that described the genotype distribution of HPV in women with different kinds of cervical lesions in Ethiopia showed the proportion of hrHPV was 77.5%. The proportion of probable HR and low-risk HPV (lrHPV) infection was found to be, 9.4% and 10.1%, respectively. The most prevalent HPV found in this review was HPV 16, followed by HPV 52, HPV 35, HPV 18 and HPV 56 [11].

Different studies suggest that the prevalence of HPV infection is affected by different factors. For instance, a cross-sectional study conducted in Tanzania among 3699 women indicated the risk of hrHPV to be high among younger women and the odds of being hrHPV positive in WWH decreased with increasing age [12]. Unmarried women (single) had also a higher risk of HPV infection in the general population, according to a study conducted in the United States [13].

Studies showed that among WWH, cervical precancerous lesions are quite prevalent. A study that assessed the magnitude of VIA positive precancerous cervical lesions among refugees in northern Ethiopia found it to be 9% [14]. Another study that assessed the prevalence of precancerous cervical lesion among WWH in southern Ethiopia showed 22.1% positivity [15]. This same study also showed that women with a lifetime history of sexually transmitted disease were 2.3 times more likely to develop precancerous cervical lesions than those who had never had sexually transmitted disease.

In Ethiopia, there are only limited studies conducted on the prevalence of hrHPV infection, even fewer studies focusing on WWH. Therefore, this study aimed to evaluate the prevalence of HPV infection in WWH, using the HPV-DNA detection test, and to assess the proportion of HPV16, 18 or “other” hrHPV genotypes distribution and associated factors in Addis Ababa, Ethiopia.

## Methods

### Study design and settings

A facility-based, retrospective record review was conducted from February 20 to March 30, 2022, in Addis Ababa, Ethiopia where pilot HPV-DNA testing was conducted. There were 18 pilot health facilities in Addis Ababa where HPV-DNA testing had been initiated in April 2021, and 16 of them were included in this study. These are high-load facilities providing antiretroviral therapy (ART) services to more than a thousand clients. This study included 6 hospitals, and 10 health centres located in different parts of Addis Ababa.

### Sample size and procedure

All WWH aged 25–49 years who gave samples for HPV testing from April 2021 up to January 2022 at the 16 health facilities were included in this study. A total of 3303 WWH were screened using HPV-DNA testing during this period and were included in this study. None were excluded.

### Data collection procedure

HPV-DNA testing was initiated at 18 pilot health facilities from April 2021 to January 2022 in Addis Ababa. Out of the health facilities, 16 were included in this study as two of the facilities joined later. These are high-load facilities providing antiretroviral therapy (ART) services to more than a thousand clients, exact data on total number of WWH was not available. Data on those who were not tested for HPV DNA is not available. The national cervical cancer screening and treatment registration logbook contained all of the screened client information, including age, marital status, education level, self-reported history of STIs (self or partner), number of births, ART status, type of visit, date and result of HPV sample collection, and VIA result of HPV Positive clients (if performed). Trained nurses who were working in the cervical cancer screening unit collected the data using a paper-based data collection tool that consisted of all the variables mentioned above. The collected data were checked for consistency and completeness before any attempt to enter, code and analyse.

### HPV-DNA testing procedure

The sample for HPV-DNA testing was collected by the client herself or a trained professional. The vaginal sample, if the client collected the sample herself, was taken in private in the health centre. The women were instructed to place half the length of the polyester swab tip in the vaginal canal and repeatedly rotate it. Afterwards to place the swab in the specimen transport buffer. The endocervical sample (specimen) was collected by inserting a cervical brush into the endocervix and rotating it three times counterclockwise in a full circle. After the specimen was collected, the cervical brush was placed in a specimen transport buffer (guanidine thiocyanate in Tris buffer) and rotated 10 times while being pushed against the wall of the transport tube. Afterwards, the specimen was sent to the facility laboratory and then transported to one of six laboratory sites within a maximum of seven days, where it was tested for HPV-DNA using the Abbott Real-Time hrHPV test. The Abbott Real-Time hrHPV test is a qualitative in vitro test for the detection of DNA from 14 hrHPV genotypes (16, 18, 31, 33, 35, 39, 45, 51, 52, 56, 58, 59, 66 and 68) in clinical specimens by polymerase chain reaction (PCR) amplification. It specifically identifies HPV genotypes 16 and 18 while concurrently detecting the other high-risk genotypes at clinically relevant infection levels. The test result was interpreted according to the manufacturer’s instructions. The results of the HPV DNA test were classified as HPV 16, HPV 18, and “other hrHPV types” or multiple hrHPV infection (any combination of the 3 results). The result was documented in the registration logbook. HPV-positive clients were then contacted by phone and told to come back for a follow-up screening test using VIA. The documented data from these screened women were used for this research.

### VIA testing procedure

VIA was performed according to the standard procedure recommended by the World Health Organisation (WHO). A bivalve speculum was inserted into the vagina, and the cervix is visualized to identify the Squamo-Columnar Junction (SCJ). Any excess mucus was cleaned using a cotton swab followed by application of 5% acetic acid on the cervix using a cotton swab, and findings became visible 1 min after application. This test was then followed by a naked eye inspection. Results of VIA were classified as negative, positive or suspicious for cervical cancer according to the International Agency for Research on Cancer (IARC) training manual. Treatment was then offered; either cryotherapy, thermal ablation or Loop Electrosurgical Excision Procedure (LEEP) based on cervical lesion size. Finally, the result was documented in the cervical cancer screening and treatment registration logbook.

### Data management and analysis

Descriptive statistics were computed and presented using frequencies and percentages. Binary logistic regression was used to analyse the association between the independent variables and HPV infection as well as cervical lesions screened through VIA. 95% confidence interval (CI) was used, and a result with a p-value < 0.05 was considered as a statistically significant association.

The basic assumption of the binary logistic regression model, multicollinearity and outliers were checked using a variance inflation factor and boxplot, respectively, and the data fulfilled the assumptions except for VIA results. Due to the low frequency of ‘unknown cervical results’ (n = 3), the three cases were removed from the analysis. ‘Suspicious for cervical cancer’, which is technically VIA positive, was merged with ‘VIA-positive’ results for the purposes of regression analysis. Finally, the multivariable binary logistic regression model was used to analyse the association between HPV positivity and the independent variables which included age, marital status, education, number of births and history of STI. Model adequacy test was done using Hosmer and Lemeshow test.

## Results

### Characteristics of study participants

A total of 3303 WWH who had given HPV samples collected from April 2021 up to January 2022 at 16 pilot health facilities were enrolled in this study. Out of these women, 46% where in the age group 40–49 years and 47.6% were married.

All study participants (*n* = 3303) were assessed for a history of STIs other than HIV and their current ART status; at the time of enrolment, 707 (21.4%) gave a positive history, and 3301 (99.9%) were taking ART (Table 1).

**Table 1:** Characteristics of enrolled women and HrHPV genotype distribution, addis ababa, ethiopia, 2021–2022 (*n* = 3303)

Characteristics	Frequency (*n*)	Percentage
Age group		
25–29 years	272	8.23%
30–39 years	1511	45.75%
40–49 years	1520	46.02%
Marital status		
Single	397	12.02%
Married	1573	47.62%
Divorced	801	24.25%
Widowed	532	16.11%
Level of education		
Illiterate	528	15.99%
Can read and write	449	13.59%
Elementary/junior	1064	32.21%
High school	863	26.13%
Tertiary school	399	12.08%
Number of Births		
No births	817	24.74%
1 birth	983	29.76%
2 births	987	29.88%
3 births and above	516	15.62%
History of STI		
Yes	707	21.40%
No	2596	78.60%
Clients’ ART status		
Yes	3301	99.94%
No	2	0.06%
hrHPV genotype distribution		
HPV not detected	2355	71.30%
HPV Positive	948	28.70%
HPV 16	145	15.30%
HPV 18	38	4.00%
Other hrHPV	695	73.30%
Multiple hrHPV	70	7.40%

### Overall and age-specific prevalence of HrHPV infection

There were 948 participating women who were tested positive for hrHPV, showing an overall prevalence of hrHPV 28.7% (95% CI: 27.2–30.3). The highest prevalence of hrHPV infection was found among women in the 25–29 age group, with an infection rate of 37.9%. Most of the women who tested positive for hrHPV were married (436 [46%]), had attended elementary school (291 [30.6%]) and had no history of STI (736 (77.6%) (Table 2).

**Table 2:** Factors associated with hrHPV-DNA positivity among enrolled women tested for hrHPV-DNA, addis ababa, ethiopia, 2021–2022 (*n* = 3303)

Variables	*p*-Value	HPV Result		COR (95% CI)	AOR (95% CI)
		Negative (*n* = 2355) n (%)	Positive (*n* = 948) n (%)		
Age in years					
25–29	< 0.001*	169 (62.13)	103 (37.87)	1.65 (1.26, 2.15)	1.68 (1.26, 2.22)
30–39	0.303	1077 (71.28)	434 (28.72)	1.09 (0.93, 1.28)	1.09(0.93,1.28)
40–49		1109 (72.96)	411 (27.04)	1	1
Marital status					
Single	0.899	270 (68.01)	127 (31.99)	1.03 (0.79, 1.33)	0.98 (0.75, 1.29)
Married	0.031*	1137 (72.28)	436 (27.72)	0.84 (0.69, 1.00)	0.81 (0.67, 0.98)
Widowed	0.015*	399 (75.00)	133 (25.00)	0.73 (0.57, 0.93)	0.733 (0.57, 0.94)
Divorced		549 (68.54)	252 (31.46)	1	1
Education					
Illiterate	0.890	373 (70.64)	155 (29.36)	1.03 (0.77, 1.37)	1.02 (0.76, 1.36)
Can read and write	0.352	305 (67.93)	144 (32.07)	1.17 (0.87, 1.56)	1.15 (0.86, 1.55)
Elementary	0.532	773 (72.65)	291 (27.35)	0.93 (0.72, 1.20)	0.92 (0.71, 1.19)
High school	0.762	620 (71.84)	243 (28.16)	0.97 (0.74, 1.26)	0.96 (0.74, 1.25)
Tertiary		284 (71.18)	115 (28.82)	1	1
Number of births					
No birth	0.498	584 (71.48)	233 (28.52)	1.04 (0.81, 1.33)	0.92 (0.71, 1.18)
1 birth	0.332	715 (72.74)	268 (27.26)	0.98 (0.77, 1.24)	0.89 (0.69, 1.13)
2 births	0.356	683 (69.20)	304 (30.80)	1.16 (0.92, 1.47)	1.12 (0.88, 1.41)
3 births and above		373 (72.29)	143 (27.71)	1	1
History of STI					
No	0.407	1860 (71.65)	736 (28.35)	0.92 (0.77, 1.10)	0.93 (0.77, 1.11)
Yes		495 (70.01)	212 (29.99)	1	1

### HrHPV genotype distribution

Out of the 984 identified hrHPV-positive results, HPV 16 accounted for 145 (15.3%), HPV 18 38 (4.0%) and other hrHPV types accounted for 695 (73.3%) (Table 1). The data showed that 70 (7.4%) women had multiple hrHPV infection. Multiple hrHPV infection is reported when the machine identifies any combination of HPV 16, HPV 18 and Other hrHPV types in a single sample.

### Factors associated with hrHPV-DNA positivity

Both bivariate and multivariable analysis was performed for age, marital status, level of education, number of births and STI, and those results in multivariate analysis were interpreted and discussed. This was done after model for adequacy showed a good fit with p-value of 0.850. Age was significantly associated with hrHPV-DNA positivity. The odds of women aged 25–29 years being infected with hrHPV were 68% higher than women aged 40–49 (AOR = 1.68, 95% CI 1.26–2.22). There was also a significant association with marital status. The odds of married women being infected with hrHPV was 19% lower as compared to women who were divorced (AOR = 0.81, 95% CI 0.67–0.98). Similarly widowed women also had 27% lower odds of having hrHPV infection as compared to divorced women (AOR = 0.73, 95% CI 0.57–0.94) (Table 2).

### VIA positivity among hrHPV-positive clients

Out of 948 women who tested positive for hrHPV infection, only 786 (83%) women came for VIA screening after being contacted via phone during our study period, though some of the remaining women may have come afterwards. The majority, which accounted for 642 (81.7%), tested VIA negative. Out of the 122 VIA-positive clients, nearly one fifth (18 [20.2%]) were between 25 and 29 years of age, followed by 30–39 years of age, which accounted for 59 (16.1%). 19 (17%) VIA-positive clients were divorced, and 23 (20%) could read and write. Overall, 80 (13.4%) patients reported no previous history of STI among VIA-positive clients (Table 3).

**Table 3:** Factors associated with VIA positivity among enrolled women who were tested positive for HPV-DNA, addis ababa, ethiopia, 2021–2022 (*n* = 783)

Variables	*P*-Value	VIA screening result			COR (95% CI)			AOR (95% CI)
			Negative (*n* = 642) *n* (%)	Positive (*n* = 141) *n* (%)				
Age in years								
25–29			70 (78.65)	19 (20.35)		1	1	
30–39	0.410		301(82.47)	64 (17.53)		0.78 (0.44,1.39)	0.78 (0.43,1.42)	
40–49	0.394		271(82.37)	58 (17.63)		0.79 (0.44,1.41)	0.76 (0.41,1.42)	
Marital status								
Single			84 (83.17)	17 (16.83)		1	1	
Married	0.590		297 (82.04)	65 (17.96)		1.08 (0.60,1.94)	1.18 (0.64, 2.18)	
Widowed	0.161		86 (76.79)	26 (23.21)		1.49 (0.76,2.95)	1.68 (0.81, 3.49)	
Divorced	0.986		175 (84.13)	33 (15.87)		0.93 (0.49,1.77)	0.99 (0.51, 1.94)	
Educational status								
Illiterate			103(84.43)	19 (15.57)		1	1	
Can read and write	0.087		88 (76.52)	27 (23.48)		1.66 (0.87,3.19)	1.79 (0.92, 3.48)	
Elementary	0.321		200 (80.00)	50 (20.00)		1.36 (0.76,2.42)	1.35 (0.75, 2.44)	
High school	0.708		172 (86.00)	28 (14.00)		0.88 (0.47,1.66)	0.88 (0.46, 1.68)	
Tertiary	0.606		79 (82.29)	17 (17.71)		1.17 (0.57,2.39)	1.21 (0.58, 2.52)	
History of STI								
No			504 (84.0)	96 (16.0)		1	1	
Yes	0.012*		138 (75.41)	45 (24.59)		1.71 (1.15,2.56)	1.69 (1.12, 2.54)	
hrHPV Genotype								
HPV 16			94 (77.69)	27 (22.31)		1	1	
HPV 18	0.414		26 (83.87)	5 (16.13)		0.67 (0.24,1.91)	0.64 (0.22, 1.86)	
Other hrHPV	0.086		485 (83.77)	94 (16.23)		0.68 (0.42,1.09)	0.65 (0.40,1.06)	
Multiple hrHPV	0.398		37 (71.15)	15 (28.85)		1.41 (0.68,2.95)	1.39 (0.65,2.96)	

### Factors associated with VIA positivity

The multivariable analysis showed that a history of STI was found to have a statistically significant relationship with the result of VIA screening. Accordingly, adjusting for other covariates, the odds of having a cervical lesion identified based on VIA screening was increased by 68.8% in women who had a history of STI as compared to women who did not have a history of STI (AOR = 1.69, 95% CI 1.12 − 2.54) (Table 3).

## Discussion

This study assessed the prevalence of hrHPV infection, the hrHPV genotype distribution, cervical lesions and associated factors in WWH living in Addis Ababa. Using a facility-based retrospective record review of women screened for cervical cancer by HPV-DNA testing and VIA, the study found that the prevalence of hrHPV infection among WWH in Addis Ababa was 27.8%. HPV16 and HPV 18 accounted for 15.3% and 4% respectively. The proportion of women infected with the 12 high-risk genotypes (31, 33, 35, 39, 45, 51, 52, 56, 58, 59, 66 and 68) other than 16 and 18, termed in the study as ‘others’, was considerably high at 73.3%. VIA screening was conducted in women who were tested positive for hrHPV to detect cervical lesion. The prevalence of VIA positive result in women who were tested positive for hrHPV was 15.5%.

The prevalence of hrHPV infection is said to differ by geographic location, socioeconomic conditions and associated illnesses, such as HIV [16]. This study found the prevalence of hrHPV infection to be 27.8%, which is higher as compared to other studies in Ethiopia. This result might reflect the fact that hrHPV infection is more prevalent among WWH than in the general population because of the inability of the host to clear the infection and reactivation of latent HPV infections due to immune suppression. For instance, a study performed in South Central Ethiopia among the rural general population found the prevalence of HPV to be 23.2% and that of hrHPV to be 20.5% [17]. Another study from Attat Hospital found the prevalence of HPV to be 17.3% and that of hrHPV to be 16% [9]. As compared to studies mentioned above, the prevalence in this study was higher, which might be due to the earlier stated higher rate of infection with HPV in WWH. Additionally, differences in test performance can be considered. However, the prevalence in this study is consistent with the 26.5% hrHPV prevalence identified in Rwanda [18]. With regard to genotype distribution, globally, the most prevalent hrHPV genotypes are HPV 16 and 18, but a study from South Central Ethiopia found that the most common hrHPV genotype was 16, followed by 35, 52, 31, 45 and 18 [17]. Whereas the most common genotypes were 16 and 18, while the remaining larger proportion were listed as others in this study.

The study showed that age group 25–29 years was significantly associated with higher odds of hrHPV-positive result than being between 40 and 49 years. This finding is consistent with multiple studies showing a higher prevalence of hrHPV infection in younger age groups, but most women will clear the infection during subsequent years. For instance, a study from South Africa showed that the prevalence of hrHPV in both HIV-positive and -negative women was the highest in the youngest age group, which was 18–25 years [19]. Another study from Kenya found that WWH under the age of 30 had the highest prevalence of hrHPV [20]. This could be attributed to risky sexual behaviors commonly observed among young adults [21, 22]. Additionally, individuals in this age group may have been recently exposed to HPV, giving their immune systems less time to clear the infection [23]. The study also showed married and widowed women were significantly associated with lower odds of hrHPV infection as compared to divorced women. This finding is also seen in other studies. For instance a study out of Italy found that never married or divorced women were factors associated with hrHPV infection [24]. Similar finding was also seen in Ghana where being single or divorced was highly associated with hrHPV infection [25].

In follow up screening, a considerable proportion (83%) of the hrHPV-positive women came back for VIA screening, and 15.5% of these women had VIA positive result. This result is similar to those of a study conducted in Amhara region, which had a prevalence of cervical lesions in 13.1% [26]. The results for cervical lesions are higher than those described in other studies, which had not initially screened for hrHPV infection before assessing for cervical lesions. For instance, in a study done in North-Western Ethiopia found the prevalence of cervical lesions among WWH to be 9.3% which is lower than the findings in this study [27]. Similarly, a study done in Amhara regional state (Woldia and Dessie Hospitals) the prevalence of cervical lesion among WWH was 9.9% [28]. A study performed in two health centres in Kenya found VIA positivity in these women to be 7%, which was lower than the results of our study [29]. The women in these studies were not initially screened for hrHPV infection which may have contributed to the lower prevalence as compared to this study.

The study also showed that a history of STI was significantly associated with having a cervical lesion diagnosed by VIA. The odds of women who had history of STI were 1.69 times more likely to have cervical lesions diagnosed by VIA than women with no history of STI. This may be because cervical precancerous lesions from hrHPV, a sexually transmitted infection, commonly co-occur with other STIs due to similar transmission methods. This finding was seen with other studies performed both in Ethiopia and in other African countries evaluating cervical lesions in WWH [15, 30] but it is worth mentioning that this study did not find a direct association between hrHPV and a history of STI. This finding may also be as a result of increased cervical inflammation that is seen with STI, studies have shown than cervical inflammation seems to contribute to an increase in false positive VIA test [31, 32].

The strength of this study was that the collected data included all women who had HPV-DNA testing done at 16 pilot health facilities during the study period and therefore enrolled a higher sample size compared to other studies conducted in Ethiopia. In addition, the pilot facilities were located in different sub-cities across Addis Ababa, which increased the representativeness of the study sample.

However, this study had some limitations, like the inability of the Abbott Real-Time hrHPV test, which was used to analyse the hrHPV genotypes, to report specific HPV genotypes other than HPV 16 and 18. The machine reports HPV 31, 33, 35, 39, 45, 51, 52 56, 58, 59, 66 and 68 as “other hrHPV genotypes” and a further differentiation of these positive tested samples in specific HPV genotypes was not possible. In addition, the findings of the study would have been better characterized if they were analysed with other independent factors such as age at first intercourse, number of sexual partners, CD4 count and viral load, but these data were not recorded in the registration logbooks used for this study.

## Conclusion

The prevalence of hrHPV infection was high (28.7%) among WWH in this study as compared to other studies performed in Ethiopia. The highest infection rate was observed in women aged 20–25 years. Notably, the prevalence of HPV 16, which has the highest carcinogenic potential, was found to be 15.3%. A high proportion of women accepted the invitation to attend VIA after receiving a positive result for hrHPV. This justifies the utilization of resources for HPV testing. A history of STI was associated with the presence of VIA abnormality and should warrant close monitoring.

## Data Availability

The datasets used and/or analyzed during the current study are available from the corresponding author on reasonable request.

## Abbreviations

AOR: Adjusted odds ratio
ART: Anti-retroviral treatment
CI: Confidence interval
COR: Crude odds ratio
DNA: Deoxyribonucleic acid
HIV: Human immunodeficiency virus
HPV: Human papillomavirus
hrHPV: High risk human papillomavirus
lrHPH: Low risk human papillomavirus
PCR: Polymerase chain reaction
SCJ: Squmo columnar junction
STI: Sexually transmitted infection
VIA: Visual inspection with acetic acid
WWH: Women with HIV
